# Development and Internal Validation of an Electronic Medical Record–Based Machine Learning Model for Frailty Prediction in Hospitalized Patients: Retrospective Cohort Study

**DOI:** 10.2196/95478

**Published:** 2026-10-28

**Authors:** Georgina Christine Laurea Szabo, Madison Bills, Matthew Williams, Annie Conway, Leesa Van Bochove, Gerry O'Callaghan

**Affiliations:** 1 SA Health, Commission on Excellence and Innovation in Health, Adelaide, South Australia, Australia; 2 Central Adelaide Local Health Network, Royal Adelaide Hospital, 1 Port Road, Adelaide, South Australia, 5000, Australia, 61 0432831520; 3 School of Public Health, Adelaide University, Adelaide, South Australia, Australia; 4 Adelaide EpiCentre, Central Adelaide Local Health Network, Adelaide, South Australia, Australia

**Keywords:** frailty, clinical prediction models, electronic medical records, clinical decision support, inpatients, supervised machine learning, allied health data, nursing documentation

## Abstract

**Background:**

Frailty is prevalent among hospitalized patients and is associated with increased risk of complications, prolonged hospitalization, higher mortality, and health care costs. Existing frailty screening tools, including the Clinical Frailty Scale (CFS) and Hospital Frailty Risk Score (HFRS), have limitations related to subjectivity, resource intensity, or reliance on retrospective data, limiting their use in routine acute care. Early identification of frailty is critical to support targeted interventions and resource allocation, yet scalable approaches for predicting frailty early during hospital admission remain limited.

**Objective:**

This paper evaluates whether routinely collected electronic medical record (EMR) data available within the first 72 hours of admission can be used to predict frailty in hospitalized patients.

**Methods:**

A retrospective cohort study was conducted using routinely collected EMR data from the Royal Adelaide Hospital (RAH), including overnight admissions with a length of stay of at least 72 hours between July 2023 and December 2024. Frailty was defined using the HFRS (score >4). Predictors were derived from EMR data available within 72 hours of admission, including demographics, medications, laboratory results, nursing assessments, and medical and allied health consult orders. Data were split into training (21,436/26,824, 80%) and test (5388/26,824, 20%) sets. Multiple machine learning models were trained, with the *F*_2_-score as the primary optimization metric. Model performance was evaluated using the *F*_2_-score, recall, area under the receiver operating characteristic curve (AUROC), calibration, and clinical utility analyses.

**Results:**

This study included 26,824 admissions, of whom 9427 (35%) were classified as frail. The final XGBoost (XGB) model achieved an *F*_2_-score of 0.755 (95% CI 0.741-0.768), an AUROC of 0.777 (95% CI 0.764-0.790), and a recall of 0.873 (95% CI 0.857-0.887) on the test dataset, with good calibration and an expected-to-observed ratio close to 1 (0.996, 95% CI 0.963-1.028). Key predictors included polypharmacy, laboratory results (including creatinine, albumin, and sodium), allied health consult orders, and nursing assessment data. Model performance was lower at 24 hours postadmission and improved by discharge.

**Conclusions:**

An EMR-based frailty prediction model using data available at 72 hours postadmission can identify patients with frailty early in their hospital stay, supporting targeted interventions and operational planning. Reduced performance in Indigenous patients highlights the need for culturally validated frailty indices and ongoing evaluation of equity in predictive modeling.

## Introduction

### Frailty in Hospitalized Patients

Patients with frailty admitted to hospital are at an increased risk of poorer outcomes, including an increased risk of hospital-acquired complications, prolonged length of stay, and higher mortality [1]. Although several validated frailty screening and assessment tools exist, frailty remains underrecognized in acute care for various reasons, including resource constraints and a lack of hospital protocols [2,3]. Early identification of frailty in the hospital setting enables targeted interventions that can improve patient outcomes and optimize resource allocation [3]. Frailty disproportionately affects certain segments of the population, including Indigenous Australians and patients with lower socioeconomic status [4,5].

The Clinical Frailty Scale (CFS) is a commonly used tool to screen for frailty; however, it relies on clinician judgment, making it subjective and resource-intensive for screening large numbers of patients [6]. The Hospital Frailty Risk Score (HFRS) [7] can be derived from ICD-10-AM (International Statistical Classification of Diseases and Related Health Problems, Tenth Revision, Australian Modification) diagnosis codes within the electronic medical record (EMR); however, as these codes are generated after discharge, the score cannot be used for early frailty identification during hospitalization.

Many frailty models are derived from diagnosis codes; however, other approaches have incorporated routinely collected clinical data, such as laboratory test results [8]. Nursing and allied health (AH) documentation are rich sources of structured data, providing insight into patients’ health and functional status, but remain underused in predictive modeling and risk stratification. Diagnoses, demographic characteristics, medications, laboratory results, procedures, and vital signs data are the most common types of structured EMR data used in frailty prediction models [9]. Nursing documentation includes assessments of mobility, malnutrition, and activities of daily living (ADLs), which are associated with frailty [10].

### Methodological Foundation

This study builds on the EMR data model and methodological approach developed by Bills et al [11], who examined whether routinely collected EMR data could be used to characterize hospital demand, care complexity, and resource use in a public quaternary teaching hospital in South Australia. In that work, hospital admissions were classified into 4 patient flow streams reflecting differing patterns of operational complexity and resource use. Of these, flow stream 3 represented a heterogeneous cohort of patients with intermediate complexity who required input from multiple disciplines and accounted for a substantial proportion of bed use, 18.6% of total admissions. Frailty is an important contributor to complexity within this cohort, given its association with functional vulnerability, prolonged hospital stays, and the need for coordinated multidisciplinary care. This study extends this EMR-based methodological framework to the related task of early frailty prediction, using routinely collected structured data available within 72 hours of admission.

### Need for Early and Scalable Frailty Identification

Currently, no validated frailty index can be readily calculated across an entire hospital early in a patient’s admission. This study aimed to develop and internally validate a frailty prediction model using routinely collected EMR data available within 72 hours of admission. The model could enable the early identification of patients with frailty for targeted clinical interventions, operational planning, and resource allocation.

### Key Objectives

Key objectives were to build a robust frailty prediction model based on the data available 72 hours after hospital admission, identify important predictors of frailty, and evaluate the model’s performance at 24 hours after admission and at discharge.

## Methods

This study was conducted and reported in accordance with the TRIPOD-AI (Transparent Reporting of a Multivariable Prediction Model for Individual Prognosis or Diagnosis-AI) guidelines for clinical prediction models using machine learning (ML) methods.

### Ethical Considerations

Approval was granted by the Central Adelaide Local Health Network (CALHN) Human Research Ethics Review Committee (2024/HRE00225), with a waiver of individual consent due to the retrospective nature and scale of the cohort. Access to EMR data was authorized by the data custodian, and data handling complied with relevant governance and privacy legislation. Data were deidentified at extraction to meet confidentiality and ethical considerations. The manuscript and supplementary materials contain no identifiable patient images. Participants did not receive any compensation for participation in the study as it involved a retrospective analysis of routinely collected data.

### Study Population

The Royal Adelaide Hospital (RAH) is an 812-bed principal referral hospital within the CALHN, providing several specialized statewide services for South Australia [12]. The study used data from the statewide EMR database, which served as the data source for model development and validation. All admissions between July 1, 2023, and December 30, 2024, were extracted.

Of 103,258 admissions during the study period, admissions with a length of stay less than 72 hours were excluded. This included day-only elective surgical admissions, which were not eligible because they did not meet the minimum 72-hour length-of-stay criterion. Patients younger than 18 years were also excluded. Overnight elective surgical admissions with a length of stay of at least 72 hours remained eligible for inclusion (26,824). The final study cohort comprised 26,824 admissions from 20,667 unique patients. Of these patients, 3862 (19%) had multiple eligible admissions during the study period. Direct age-stratified frailty rates were calculated to compare frailty between Indigenous and non-Indigenous populations.

The EMR was used as a data source because of its comprehensive capture of inpatient data, including documentation from multiple disciplines, including administrative, medical, nursing, and AH.

Use of a common statewide EMR may support generalizability and implementation of the modeling approach at other South Australian public hospitals; however, the cohort was drawn from a single quaternary hospital and requires external validation before broader generalizability can be established.

### Data Extraction

Data were extracted from the EMR database. Extraction logic was based on hospital admission identifiers, admission and discharge timestamps, and clinical documentation tables relevant to laboratory, medication, nursing, and consult order data. Extracted data were deidentified prior to analysis. Data underwent initial validation to confirm completeness and consistency with source records, and duplicate or implausible entries (eg, negative timestamps or null admission identifiers) were excluded. Each hospital admission was treated as an independent entity, and only the first 72 hours of data from each admission were used to derive predictor variables. Separate indicator sets were derived for the primary analysis of 72 hours and the alternate 24 hours and discharge time points for evaluation.

### Data Processing

Data processing and analysis were conducted using Python (version 3.12.3). HFRS scores were generated for each visit using ICD-10-AM codes assigned after discharge, characterizing visits as frail (HFRS >4; intermediate or high risk) or not frail (HFRS <5; low risk), which was used as the outcome variable for model training. ICD-10-AM codes are standardized and used across all public hospitals in South Australia, so they are generated consistently across demographic groups. The HFRS, derived from these codes, ensures consistent outcome assessment across sociodemographic groups in this dataset.

Categorical variables were one-hot encoded using *OneHotEncoder* from scikit-learn (version 1.4.2), which converts categorical variables into a binary format, with each category represented by a separate column with values of 0 or 1. Missing values were replaced with an “unknown” category during the one-hot encoding process to avoid arbitrary assignment. Mean imputation was used to handle missing numeric values under the assumption of missing at random, and the numeric variables were subsequently scaled using *StandardScaler* from scikit-learn. Data quality checks including profiling for missing values were conducted, with variables with high missingness (threshold >30% missing) being excluded as model predictors to reduce the risk of unreliable model estimates. The variables excluded based on missingness were previous clinical service admitted under, previous length of stay, MRI in the first 72 hours, and falls history. Model preprocessing was relatively consistent across demographic groups, suggesting that missingness was likely random.

### Sample Size Calculation

When developing a multivariate prediction model, the sample size must be sufficient to ensure reliable estimation, considering both the number of admissions and outcome events in relation to the number of predictors included [13]. The minimum sample size was calculated using the *pmsampsize* function from the R package *pmsampsize* developed by Joie Ensor (version 1.1.3) for binary prediction model development. The sample size was determined to be 1451, with a minimum of 508 events (patients with frailty), based on the 40 input parameters generated from data processing, a frailty prevalence of 35% (9,427/26,824) from the dataset, and an anticipated C-statistic of 0.78. The anticipated C-statistic was based on a recent hospital-admission frailty prediction study by Chou et al [14], which used routinely available hospital clinical data to train supervised ML models for identifying frailty in older inpatients with acute illness. The final cohort size substantially exceeded the calculated minimum, ensuring sufficient power to reliably address the research question.

### Modeling Approach

Potential model predictors were identified through clinician workshops and frailty risk factors from the literature [8,15]. Predictors were categorized into 5 groups: medication-related, laboratory test results, AH and medical consult orders, nursing assessment scores, and demographics. All predictors were derived from data available within 72 hours of hospital admission. A complete list of model predictors is available in Multimedia Appendix 1.

The 72-hour prediction window was selected to balance clinical actionability with data completeness. Although earlier prediction may allow more time for clinical intervention, key predictors such as laboratory results and AH and nursing assessments are less consistently available in the first 24 hours than demographic variables. By 72 hours, these multidisciplinary EMR data are more complete while still being available early enough to support targeted interventions during the admission.

As patients could have multiple admissions during the period, the data were split at the patient level to avoid data leakage from the same patient appearing in both the training and test datasets. The data were split into 80% (21,436/26,824) training and 20% (9427/26,824) test datasets using *train_test_split* from scikit-learn. All model training, model selection, threshold tuning, and reported performance metrics were generated using this patient-level split.

Four classification models were trained to predict the HFRS frailty grouping at discharge based on data available within 72 hours of hospital admission, including XGBoost (XGB), random forest, logistic regression, and support vector machine (SVM) models. These models were chosen based on their established use in prior ML frailty modeling studies [16]. The random forest, logistic regression, and SVM models were all from the scikit-learn library, and the *XGBClassifier* from the XGB library (version 3.1.1) was used to train the XGB model. The *F*_2_-score was chosen as the objective function for hyperparameter and threshold tuning. The *F*_2_-score, which weights recall higher than precision, was chosen to prioritize identifying patients with frailty while still maintaining reasonable precision in model predictions. Hyperparameter tuning was performed using cross-validation with the *GridSearchCV* function from scikit-learn on the training dataset. Predicted probabilities were generated using *predict_proba* from scikit-learn. The final 72-hour XGB model and selected classification threshold were then applied without retraining to predictor sets reconstructed using data available at 24 hours after admission and at discharge. These analyses were used to assess how model performance varied according to the amount of clinical information available at different stages of admission.

### Threshold Sensitivity Testing

A threshold sensitivity analysis was performed on the 72-hour training data to select the final classification threshold. *F*_2_-score, recall, and precision were assessed across thresholds from 0.10 to 0.50 on the training data. The final classification threshold was based on the optimal *F*_2_-score, which is consistent with the study aim of prioritizing frailty detection while limiting false positives. The selected threshold was then applied to the 72-hour training and test datasets and to the 24-hour and discharge predictor sets without further threshold tuning.

### Model Assessment

Model performance was assessed using discrimination, classification, and calibration metrics. The *F*_2_-score was selected as the primary evaluation metric because it weights recall higher than precision, consistent with the aim of minimizing missed frailty cases in an imbalanced dataset. The scikit-learn package was the source of all evaluation functions, unless otherwise specified. Receiver operator curves (ROCs) were generated for the training and test datasets using *roc_curve*, and area under the receiver operating characteristic curve (AUROC) was calculated using *roc_auc_score*. Classification metrics included *F*_2_-score, recall, precision, specificity, positive predictive value (PPV), and negative predictive value (NPV). Overall model performance was assessed using the Brier score, and calibration was assessed using the ratio of the expected number of cases predicted by the frailty model to the observed number of cases (expected-to-observed [E/O] ratios).

Bootstrapped 95% CIs were calculated for *F*_2_-score, recall, precision, specificity, AUROC, Brier score, and E/O ratios to quantify uncertainty around point estimates. The same evaluation metrics were calculated for the primary 72-hour test dataset and for the 24-hour and discharge predictor sets to compare model performance across admission time points. Subgroup analyses were performed by sex and Indigenous status to evaluate model bias across demographic groups.

The final prediction model comprised a preprocessing pipeline and an XGB classifier implemented in Python (version 3.12.3) using the scikit-learn and XGB libraries. Owing to ethics, governance, and data security restrictions associated with the underlying EMR data, the full model object and analytical code are not publicly available. The analytical code, preprocessing pipeline specifications, and model artifacts may be made available upon reasonable request, subject to approvals.

### Feature Importance

Feature importance was then assessed for the final 72-hour XGB model using Shapley additive explanations (SHAP) values generated by *TreeExplainer* in the SHAP library (version 0.46) to assess how each variable contributes to the overall performance of the model [17]. XGB was the final chosen model due to its superior performance and interpretable feature importance. Interpretable features are particularly important in health care, where explainability can increase end-user trust [18].

### Clinical Utility Analysis

Clinical utility was assessed using standard decision curve analysis in the test dataset. Decision curve analysis was used to estimate the net benefit of using the final XGB model to identify patients at increased risk of frailty across threshold probabilities from 0.10 to 0.50. The analysis used predicted probabilities from the final XGB model without recalibration. Net benefit was compared with the default strategies of treating all patients as frail and treating no patients as frail. In this context, a positive model prediction was interpreted as identifying a patient for potential frailty-related clinical review or intervention, although a specific implementation pathway was not prespecified.

Net benefit was calculated as the proportion of true positives minus the proportion of false positives weighted by the odds of the threshold probability. The selected operating threshold of 0.20 was not clinically prespecified; it was selected from threshold sensitivity testing to prioritize the *F*_2_-score while limiting false-positive classifications. Results at this threshold were therefore interpreted as performance at the selected statistical operating point rather than as evidence of an established clinical decision threshold. Uncertainty in net benefit estimates was quantified using 1000 nonparametric bootstrap resamples at the admission level, with 95% CIs derived from the bootstrap distribution.

## Results

### Study Population

The flow of patient exclusions is shown in Figure 1. The characteristics of the study population are detailed in Table 1. Age-stratified frailty rates differed between Indigenous and non-Indigenous patients (Table 2). In non-Indigenous patients, frailty increased progressively with age, rising from 19% (249/1289) in those aged 18 to 30 years to 54% (774/1431) in those aged 91 years and older. In Indigenous patients, frailty rates did not increase consistently across age groups, although the interpretation of older age groups is limited by small denominators. The highest observed frailty rate was in Indigenous patients aged 81 to 90 years (11/17, 65%), while rates were lower in those aged 71 to 80 years (35/121, 29%) and 91 years and over (1/3, 33%). These findings indicate different observed age-frailty profiles between Indigenous and non-Indigenous patients in the study cohort, with estimates in older Indigenous age groups likely to be unstable.

**Figure 1.**
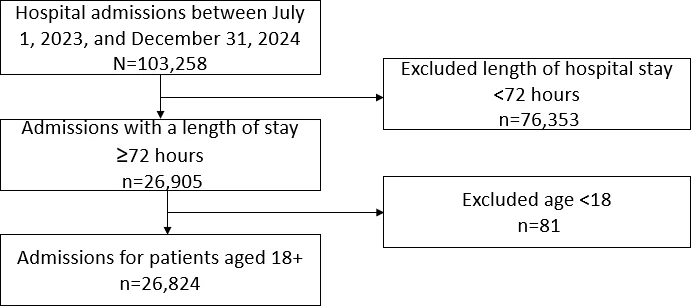
Study inclusion criteria.

**Table 1.** Study population (N=26,824).

Characteristic		Frail (n=9427)	Not frail (n=17,397)
Frailty status, mean (SD)		71 (17.22)	63 (18.87)
Age (years), n (%)			
18‐30		304 (3)	1262 (7)
31‐40		377 (4)	1397 (8)
41‐50		575 (6)	1803 (10)
51‐60		1085 (12)	2680 (15)
61‐70		1688 (18)	3473 (20)
71‐80		2259 (24)	3595 (21)
81‐90		2356 (25)	2510 (14)
Greater than 90		783 (8)	677 (4)
Length of stay (days), mean (SD)		14.5 (17.09)	8.9 (9.72)
Sex, n (%)^a^			
Female		4348 (37)	7434 (63)
Male		5077 (34)	9962 (66)
Indeterminate/unknown		2 (67)	1 (33)
Indigenous status, n (%)^a^			
Indigenous (Aboriginal and/or Torres Strait Islander)		531 (29)	1318 (71)
Non-Indigenous		8700 (36)	15,685 (64)
Unknown		196 (33)	394 (67)
Medication count at 72 hours, mean (SD)		15.6 (6.55)	12.5 (6.03)
Number of blood tests ordered at 72 hours, mean (SD)		11.4 (4.82)	9.7 (4.44)
Number of allied health consult orders at 72 hours, mean (SD)		2.3 (1.08)	1.8 (0.97)
Number of medical consult orders at 72 hours, mean (SD)		1.5 (0.81)	1.3 (0.65)

a Percentages for sex and Indigenous status represent the proportion within each category classified as frail or not frail.

**Table 2.** Age-stratified frailty prevalence by Indigenous status (N=9427).

Characteristic		Indigenous	Non-Indigenous	Unknown
Age (years), n/N (%)^a^				
18‐30		49/241 (20)	249/1289 (19)	6/36 (17)
31‐40		95/346 (28)	273/1390 (20)	9/38 (24)
41‐50		91/373 (24)	472/1952 (24)	12/53 (23)
51‐60		132/410 (32)	924/3255 (28)	29/100 (29)
61‐70		117/338 (35)	1531/4702 (33)	40/121 (33)
71‐80		35/121 (29)	2180/5609 (39)	44/124 (36)
81‐90		11/17 (65)	2297/4757 (48)	48/92 (52)
Greater than 90		1/3 (33)	774/1431 (54)	8/26 (31)

a Age group–specific frailty rates are reported.

### Modeling Approach

The development and test datasets were broadly similar with respect to demographics, frailty prevalence, and key predictor distributions (Table 3).

The best model parameters derived from hyperparameter tuning are shown in Table 4. *F*_2_-score optimization resulted in the best parameters being model__colsample_bytree=0.8, model__learning_rate=0.1, model__max_depth=5, model__n_estimators=200, and model__subsample=0.8.

**Table 3.** Comparison of development and evaluation datasets.

Characteristic	Training	Test
Admissions, n	21,436	5388
Frail, n (%)	7541 (35)	1886 (35)
Age (years), mean (SD)	66.0 (18.7)	65.4 (18.7)
Female, n (%)	9511 (44)	2271 (42)
Indigenous status, n (%)	1443 (7)	406 (8)
Medication count at 72 hours, mean (SD)	13.6 (6.4)	13.6 (6.3)
Number of blood tests ordered at 72 hours, mean (SD)	10.2 (4.6)	10.3 (4.7)
Number of allied health consult orders at 72 hours, mean (SD)	2.0 (1.0)	2.0 (1.0)
Number of medical health consult orders at 72 hours, mean (SD)	1.4 (0.7)	1.4 (0.8)

**Table 4.** Best model parameters derived from hyperparameter tuning.

Parameter	Value
model__colsample_bytree	0.8
model__learning_rate	0.1
model__max_depth	5
model__n_estimators	200
model__subsample	0.8

XGB showed the strongest overall performance across the four candidate models, as shown in Table 5. Discrimination was similar across models, with AUROC values ranging from 0.755 (95% CI 0.741‐0.769) for random forest to 0.777 (95% CI 0.764-0.790) for XGB. XGB had the highest *F*_2_-score (0.755, 95% CI 0.741‐0.768), substantially exceeding logistic regression (0.492, 95% CI 0.470‐0.512), random forest (0.421, 95% CI 0.400‐0.443), and SVM (0.486, 95% CI 0.464‐0.507). Recall was also highest for XGB (0.873, 95% CI 0.857‐0.887), compared with 0.463 (95% CI 0.441‐0.484) for logistic regression, 0.385 (95% CI 0.365‐0.408) for random forest, and 0.455 (95% CI 0.433‐0.476) for SVM. This indicates that XGB identified a substantially greater proportion of patients with frailty and was less likely to miss frailty cases. Brier score was lowest for XGB (0.181, 95% CI 0.175‐0.186), although differences between models were small. XGB was therefore selected as the final model for subsequent evaluation.

**Table 5.** Performance of candidate machine learning models for frailty prediction on the test dataset.

Model	*F*_2_-score (95% CI)	Recall (95% CI)	AUROC^a^ (95% CI)	Brier score (95% CI)
Logistic regression	0.492 (0.470‐0.512)	0.463 (0.441‐0.484)	0.768 (0.755‐0.781)	0.183 (0.178‐0.189)
Random forest	0.421 (0.400‐0.443)	0.385 (0.365‐0.408)	0.755 (0.741‐0.769)	0.189 (0.185‐0.193)
SVM^b^	0.486 (0.464‐0.507)	0.455 (0.433‐0.476)	0.760 (0.746‐0.774)	0.186 (0.180‐0.192)
XGB^c^	0.755 (0.741‐0.768)	0.873 (0.857‐0.887)	0.777 (0.764‐0.790)	0.181 (0.175‐0.186)

a AUROC: area under the receiver operating characteristic curve.

b SVM: support vector machine.

c XGB: XGBoost.

### Threshold Sensitivity Testing

Threshold sensitivity testing showed the expected trade-off between recall and precision as the classification threshold increased (Table 6). Lower thresholds produced higher recall but lower precision and specificity, while higher thresholds improved precision and specificity at the cost of missed frailty cases. The threshold of 0.20 was selected for the final model, as it had the best *F*_2_-score of 0.802 (95% CI 0.796‐0.808). This threshold was consistent with the study objective of prioritizing frailty detection while retaining moderate precision (0.519, 95% CI 0.510‐0.528) and specificity (0.533, 95% CI 0.525‐0.541).

**Table 6.** Threshold sensitivity analysis for the final XGBoost frailty prediction model.

Threshold	*F*_2_-score (95% CI)	Recall (95% CI)	Precision (95% CI)	Specificity (95% CI)	Predicted positive, n	Predicted negative, n
0.1	0.774 (0.768‐0.779)	0.985 (0.982‐0.988)	0.416 (0.408‐0.423)	0.250 (0.243‐0.257)	17,850	3586
0.15	0.795 (0.789‐0.800)	0.962 (0.958‐0.966)	0.469 (0.461‐0.477)	0.410 (0.402‐0.418)	15,454	5982
0.2	0.802 (0.796‐0.808)	0.928 (0.922‐0.934)	0.519 (0.510‐0.528)	0.533 (0.525‐0.541)	13,488	7948
0.25	0.798 (0.791‐0.804)	0.889 (0.882‐0.896)	0.566 (0.557‐0.574)	0.630 (0.621‐0.637)	11,851	9585
0.3	0.787 (0.780‐0.794)	0.848 (0.839‐0.856)	0.611 (0.602‐0.620)	0.707 (0.700‐0.715)	10,458	10,978
0.35	0.771 (0.763‐0.779)	0.805 (0.796‐0.815)	0.658 (0.648‐0.668)	0.773 (0.766‐0.780)	9225	12,211
0.4	0.741 (0.732‐0.750)	0.751 (0.741‐0.761)	0.703 (0.693‐0.713)	0.828 (0.822‐0.835)	8049	13,387
0.45	0.704 (0.694‐0.713)	0.694 (0.684‐0.705)	0.747 (0.737‐0.757)	0.872 (0.867‐0.878)	7005	14,431
0.5	0.664 (0.653‐0.674)	0.638 (0.626‐0.649)	0.792 (0.782‐0.803)	0.909 (0.905‐0.914)	6068	15,368

### Model Assessment

The ROCs shown in Figure 2, generated for the test and training datasets, showed good discrimination between the classes, with an AUROC for the training dataset of 0.874 (95% CI 0.869‐0.879). The AUROC for the test dataset was 0.777 (95% CI 0.764‐0.790), which still indicates reasonable discrimination.

**Figure 2.**
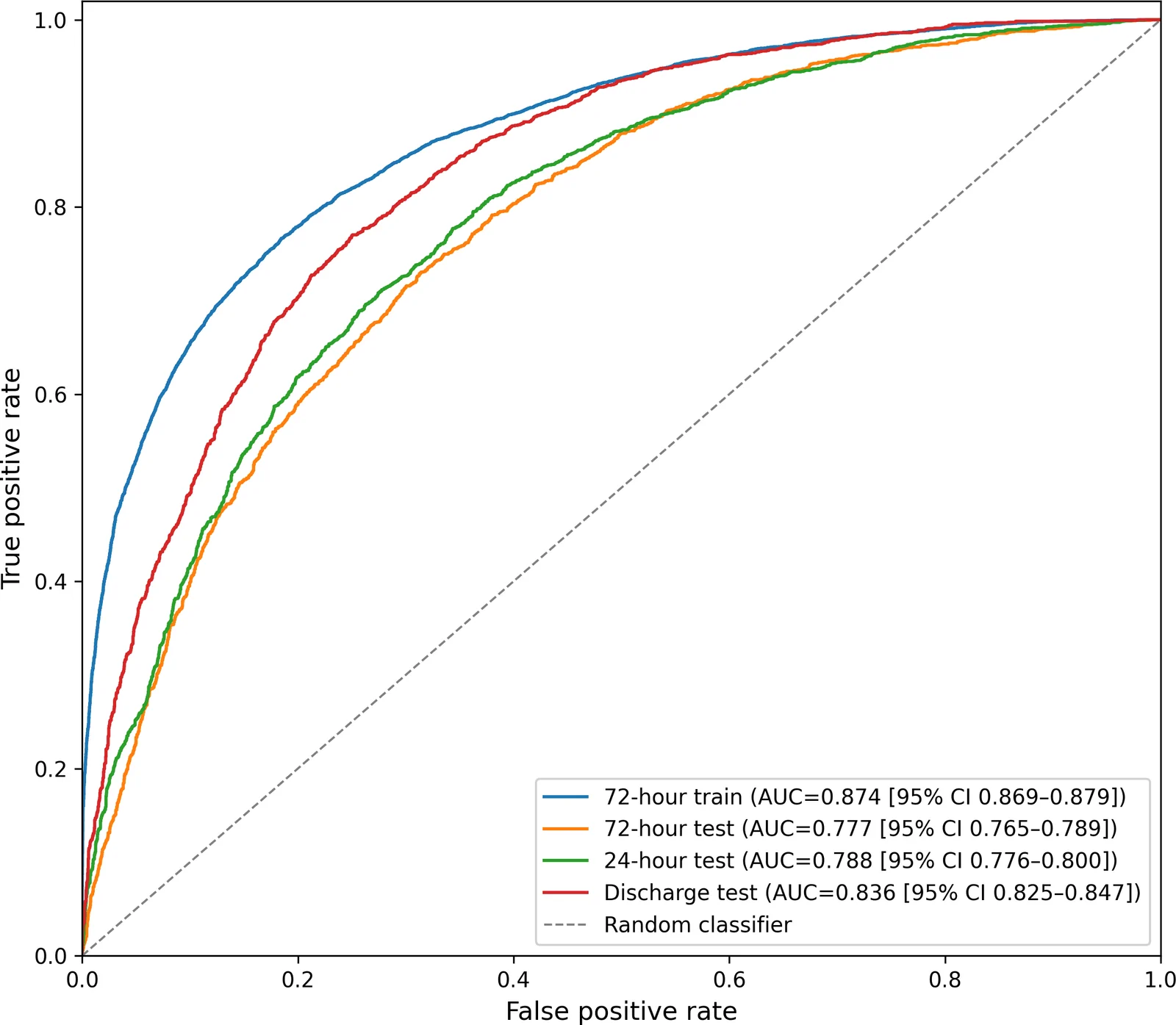
Receiver operator curves for training, test, 24-hour validation, and discharge validation datasets. AUC: area under the receiver operating characteristic curve.

Model discrimination and classification performance across training, test, 24-hour, and discharge datasets is shown in Table 7. The *F*_2_-score was 0.802 (95% CI 0.796‐0.808) for the training dataset and 0.755 (95% CI 0.741‐0.768) for the test dataset, with corresponding recall values of 0.928 (95% CI 0.922‐0.934) and 0.873 (95% CI 0.857‐0.887), respectively. Specificity was similar between the training and test datasets at 0.533 (95% CI 0.525‐0.541) and 0.511 (95% CI 0.493‐0.528), respectively. PPV was moderate in both datasets (0.519, 95% CI 0.510‐0.528 and 0.490, 95% CI 0.473‐0.506), while NPV remained high (0.932, 95% CI 0.926‐0.937 and 0.882, 95% CI 0.867‐0.895). These results indicate that the model retained high sensitivity for frailty detection, with a lower probability of false-negative classification than false-positive classification.

**Table 7.** Discrimination and classification performance of the final XGBoost frailty prediction model across prediction time points.

Dataset	*F*_2_-score (95% CI)	Recall (95% CI)	AUROC^a^ (95% CI)	Specificity (95% CI)	PPV^b^ (95% CI)	NPV^c^ (95% CI)^c^
Training	0.802 (0.796‐0.808)	0.928 (0.922‐0.934)	0.874 (0.869‐0.879)	0.533 (0.525‐0.541)	0.519 (0.510‐0.528)	0.932 (0.926‐0.937)
Test	0.755 (0.741‐0.768)	0.873 (0.857‐0.887)	0.777 (0.764‐0.790)	0.511 (0.493‐0.528)	0.490 (0.473‐0.506)	0.882 (0.867‐0.895)
24-hour test	0.742 (0.725‐0.756)	0.827 (0.809‐0.844)	0.785 (0.772‐0.797)	0.597 (0.581‐0.612)	0.525 (0.507‐0.541)	0.865 (0.851‐0.879)
Discharge test	0.796 (0.785‐0.806)	0.957 (0.947‐0.965)	0.835 (0.824‐0.846)	0.433 (0.416‐0.450)	0.476 (0.460‐0.492)	0.949 (0.938‐0.960)

a AUROC: area under the receiver operating characteristic curve.

b PPV: positive predictive value.

c NPV: negative predictive value.

Calibration was acceptable for both the training and test datasets, with E/O ratios close to 1.0 and CIs crossing 1.0 (Table 8). As shown in Table 8, the training dataset showed slight overestimation (E/O ratio 1.000, 95% CI 0.986‐1.015), while the test dataset showed slight underestimation (E/O ratio 0.996, 95% CI 0.963‐1.028). The calibration plot for the test dataset is shown in Figure 3. The Brier score was 0.136 (95% CI 0.134‐0.139) for the training dataset and 0.181 (95% CI 0.175‐0.186) for the test dataset, supporting reasonable overall probability accuracy.

**Table 8.** Calibration and overall performance of the final XGBoost frailty prediction model across prediction time points.

Dataset	Brier score (95% CI)	E/O ratio^a^ (95% CI)^a^
Training	0.136 (0.134‐0.139)	1.000 (0.986‐1.015)
Test	0.181 (0.175‐0.186)	0.996 (0.963‐1.028)
24-hour test	0.181 (0.175‐0.187)	0.826 (0.801‐0.852)
Discharge test	0.158 (0.153‐0.163)	1.123 (1.092‐1.157)

a E/O ratio: expected-to-observed ratio.

**Figure 3.**
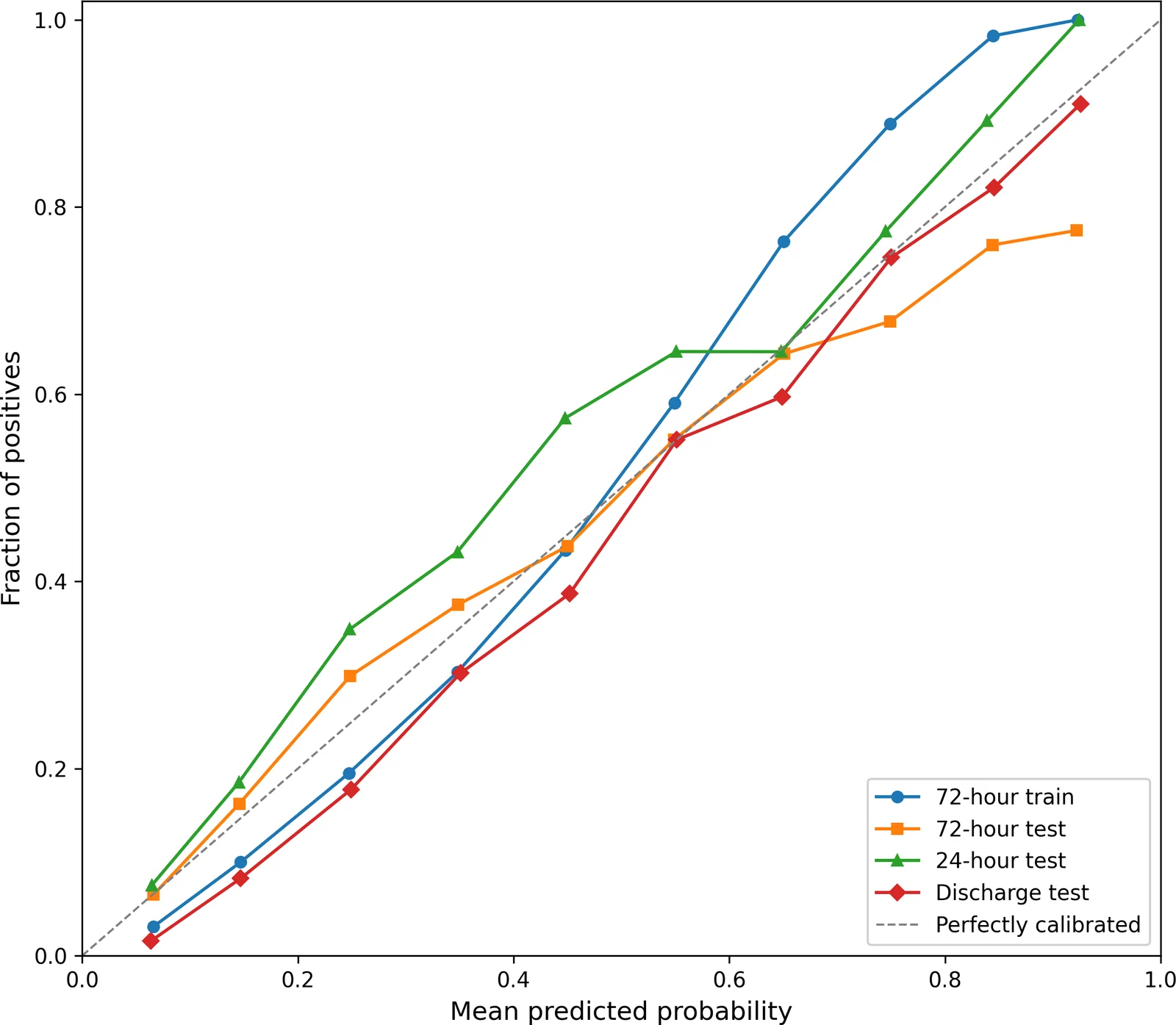
Calibration curves for the train, test, 24-hour validation, and discharge validation datasets.

At 24 hours, the *F*_2_-score and recall were lower than in the 72-hour test dataset (0.742, 95% CI 0.725‐0.756 and 0.827, 95% CI 0.809‐0.844), although specificity and PPV were higher (0.597, 95% CI 0.581‐0.612 and 0.525, 95% CI 0.507‐0.541). NPV remained high at 0.865 (95% CI 0.851‐0.879). The 24-hour model underestimated frailty, with an E/O ratio of 0.826 (95% CI 0.801‐0.852). At discharge, recall increased to 0.957 (95% CI 0.947‐0.965) and NPV to 0.949 (95% CI 0.938‐0.960), but specificity decreased to 0.433 (95% CI 0.416‐0.450) and PPV to 0.476 (95% CI 0.460‐0.492), reflecting more positive classifications. The discharge model overestimated frailty, with an E/O ratio of 1.123 (95% CI 1.092‐1.157).

Model discrimination and classification performance metrics across sex and Indigenous status are shown in Table 9. Female participants had slightly higher *F*_2_-score and recall values (0.769, 95% CI 0.749‐0.788 and 0.887, 95% CI 0.865‐0.909) compared with male participants (0.744, 95% CI 0.726‐0.764 and 0.862, 95% CI 0.842‐0.883). Male participants demonstrated higher specificity (0.533, 95% CI 0.511‐0.555 vs 0.477, 95% CI 0.450‐0.504) and NPV (0.885, 95% CI 0.868‐0.903 vs 0.876, 95% CI 0.854‐0.900), whereas female participants had higher PPV (0.502, 95% CI 0.477‐0.527 vs 0.480, 0.458‐0.503). AUROC was marginally lower in female participants (0.771, 95% CI 0.753‐0.790 vs 0.780, 95% CI 0.765‐0.797), with a higher Brier score (0.187, 95% CI 0.179‐0.196 vs 0.176, 95% CI 0.169‐0.183; Tables 9 and 10). Calibration was acceptable for both groups, with E/O ratios close to 1.0 and CIs crossing 1.0, indicating slight underestimation in female participants (0.981, 95% CI 0.935‐1.031) and slight overestimation in male participants (1.007, 95% CI 0.965‐1.050; Table 10).

**Table 9.** Discrimination and classification performance of the final XGBoost frailty prediction model by subgroup.

Subgroup	*F*_2_-score (95% CI)	Recall (95% CI)	AUROC^a^ (95% CI)	Specificity (95% CI)	PPV^b^ (95% CI)	NPV^c^ (95% CI)
Female	0.769 (0.749‐0.788)	0.887 (0.865‐0.909)	0.771 (0.753‐0.790)	0.477 (0.450‐0.504)	0.502 (0.477‐0.527)	0.876 (0.854‐0.900)
Male	0.744 (0.726‐0.764)	0.862 (0.842‐0.883)	0.780 (0.765‐0.797)	0.533 (0.511‐0.555)	0.480 (0.458‐0.503)	0.885 (0.868‐0.903)
Indigenous	0.723 (0.664‐0.776)	0.848 (0.784‐0.911)	0.798 (0.749‐0.846)	0.612 (0.558‐0.668)	0.455 (0.389‐0.520)	0.914 (0.874‐0.949)
Non-Indigenous	0.757 (0.743‐0.770)	0.874 (0.860‐0.890)	0.774 (0.761‐0.787)	0.501 (0.485‐0.518)	0.492 (0.476‐0.510)	0.878 (0.864‐0.893)

a AUROC: area under the receiver operating characteristic curve.

b PPV: positive predictive value.

c NPV: negative predictive value.

**Table 10.** Calibration and overall performance of the final XGBoost frailty prediction model by subgroup.

Subgroup	Brier score (95% CI)	E/O ratio^a^ (95% CI)
Female	0.187 (0.179‐0.196)	0.981 (0.935‐1.031)
Male	0.176 (0.169‐0.183)	1.007 (0.965‐1.050)
Indigenous	0.152 (0.132‐0.172)	1.009 (0.885‐1.168)
Non-Indigenous	0.183 (0.177‐0.189)	0.995 (0.961‐1.026)

a E/O ratio: expected-to-observed ratio.

Patients who identified as Indigenous had a lower *F*_2_-score (0.723, 95% CI 0.664‐0.776) and recall (0.848, 95% CI 0.784‐0.911) compared to patients who identified as non-Indigenous (*F*_2_-score 0.757, 95% CI 0.743‐0.770, recall 0.874, 95% CI 0.860‐0.890). Specificity was higher in Indigenous patients (0.612, 95% CI 0.558‐0.668 vs 0.501, 95% CI 0.485‐0.518), with lower PPV (0.455, 95% CI 0.389‐0.520 vs 0.492, 95% CI 0.476‐0.510) and higher NPV (0.914, 95% CI 0.874‐0.949 vs 0.878, 95% CI 0.864‐0.893). AUROC was higher for Indigenous patients (0.798, 95% CI 0.749‐0.846 vs 0.774, 95% CI 0.761‐0.787), while the Brier score was lower (0.152, 95% CI 0.132‐0.172 vs 0.183, 95% CI 0.177‐0.189), suggesting better overall probability accuracy despite poorer threshold-dependent classification performance. These findings highlight that probability-based measures and threshold-dependent classification metrics may provide different perspectives on model performance across subgroups.

As shown in Table 10, calibration was acceptable in both groups, with E/O ratios close to 1.0 and overlapping CIs (0.995, 95% CI 0.961‐1.026 vs 1.009, 95% CI 0.885‐1.168).

### Feature Importance

Figure 4 shows the 25 most important features determined by mean absolute SHAP values. The maximum serum creatinine level was the most important feature, followed by the maximum Braden pressure injury score and number of medications. This was followed by the number of blood tests ordered, minimum sodium level, whether a speech pathology consult order was placed, and independence in dressing as an ADL.

**Figure 4.**
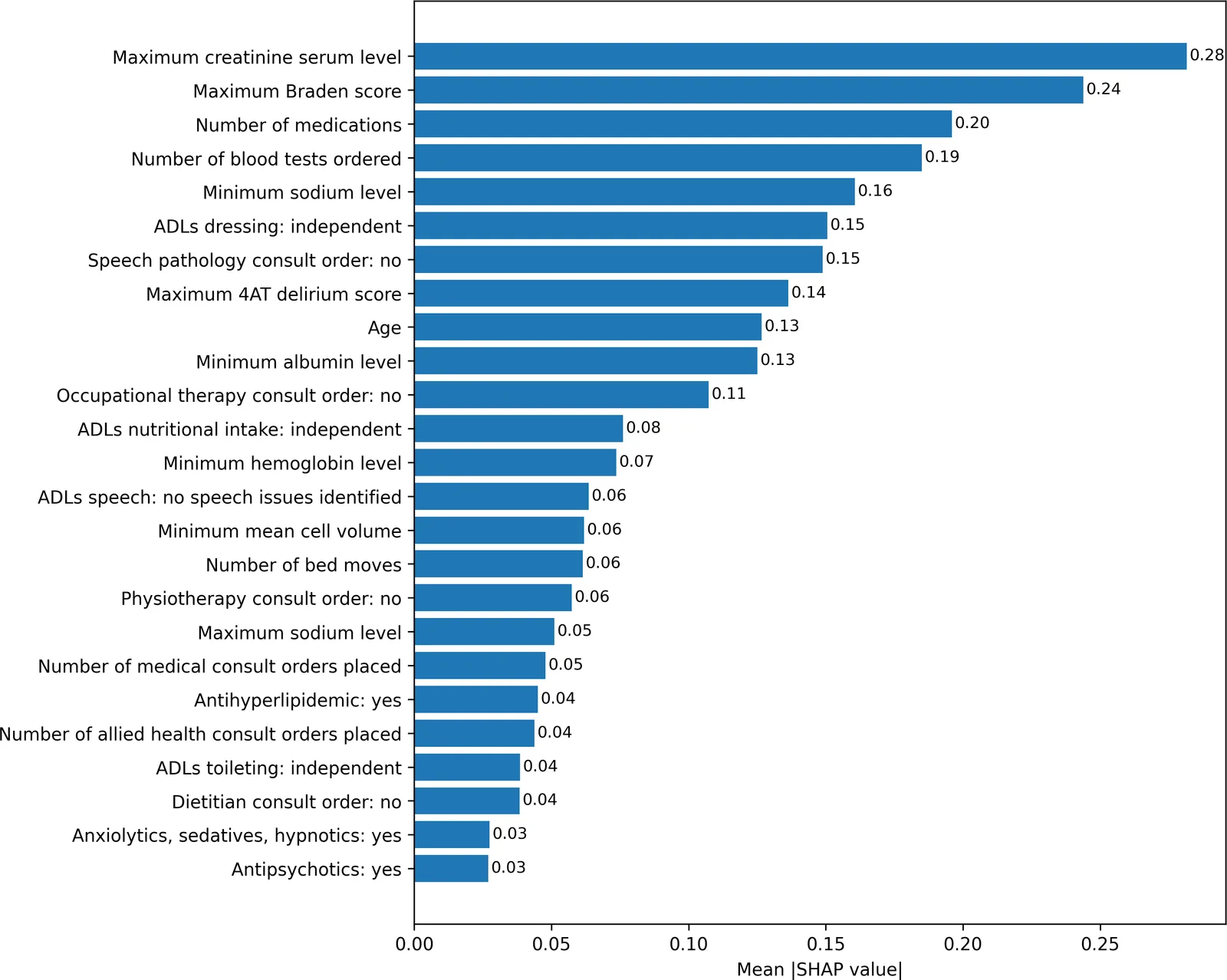
Top 25 features based on mean absolute Shapley additive explanation (SHAP) values. 4AT: delirium and cognitive impairment 4-item assessment test; ADLs: activities of daily living.

### Clinical Utility Analysis

Decision curve analysis showed that the final XGB model provided positive net benefit across all evaluated threshold probabilities from 0.10 to 0.50 (Table 11; Figure 5). The model exceeded the treat-all strategy from a threshold probability of 0.10, indicating greater clinical utility than applying frailty-related clinical review or intervention to all patients across the evaluated threshold range.

At the operating threshold of 0.20, the model achieved a net benefit of 0.224 (95% CI 0.210‐0.237). This corresponded to identifying 62% (n=3360) of admissions for potential frailty-related clinical review or intervention and a net reduction of 37.5 interventions per 100 patients compared with a treat-all strategy. Net benefit decreased as the threshold probability increased, from 0.282 at a threshold of 0.10 to 0.081 at a threshold of 0.50, while the proportion of admissions identified for review or intervention decreased from 82.9% (n=4469) to 27.9% (n=1505).

These findings suggest that the model may provide clinical utility for prioritizing frailty-related review among hospitalized patients, particularly where the acceptable threshold for action is between 0.10 and 0.30. Because no clinical risk threshold or implementation pathway was prespecified, results should be interpreted as evidence of potential utility rather than as a definitive clinical decision rule.

**Table 11.** Decision curve analysis of the final XGBoost frailty prediction model.

Threshold	Net benefit^a^ (95% CI)	Admissions identified for review^b^ (N=5388), n (%)	Net reduction per 100 admissions
0.1	0.282 (0.269‐0.294)	4469 (83)	16.6
0.15	0.253 (0.240‐0.267)	3858 (72)	27.7
0.2	0.224 (0.210‐0.237)	3360 (62)	37.5
0.25	0.197 (0.183‐0.211)	2966 (55)	44.7
0.3	0.168 (0.154‐0.182)	2614 (49)	51.2
0.35	0.142 (0.128‐0.156)	2328 (43)	56.5
0.4	0.123 (0.109‐0.137)	2028 (38)	62
0.45	0.100 (0.086‐0.113)	1757 (33)	67.5
0.5	0.081 (0.068‐0.095)	1505 (28)	72.1

a Net benefit was estimated using decision curve analysis on the test dataset.

b Admissions identified for review refers to admissions classified as positive at each threshold probability.

**Figure 5.**
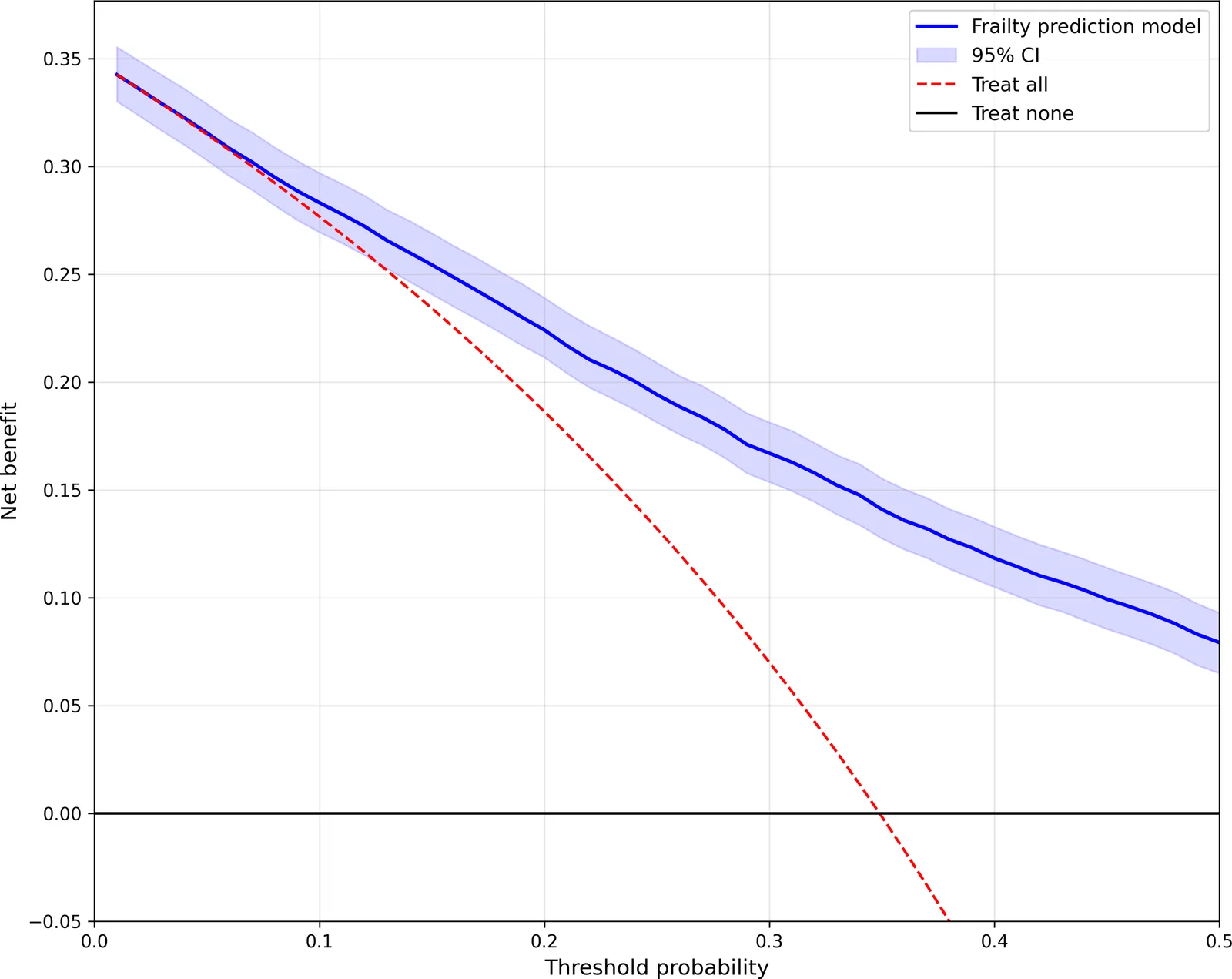
Clinical utility of the final XGBoost frailty prediction model using decision curve analysis.

## Discussion

### Principal Findings

This study aimed to develop and internally validate a frailty prediction model for hospital inpatients using routinely collected EMR data available within 72 hours of admission. The final XGB model demonstrated good discrimination (*F*_2_-score 0.755, 95% CI 0.741‐0.768; recall 0.873, 95% CI 0.857‐0.887) and calibration on the test dataset. Key predictors included polypharmacy, laboratory results (creatinine, albumin, and sodium), AH consult orders, and nursing assessment data. Model performance improved as more clinical information became available during admission but was reduced for Indigenous patients, potentially highlighting limitations of the HFRS in this subgroup. These findings indicate that early frailty prediction using routine EMR data is feasible and may inform both clinical decision-making and operational planning, though further external validation and attention to equity are required.

The performance of the present model was broadly comparable to previously published frailty prediction models. Chou et al [14] developed a ML frailty detection model using 21 variables and a 6-month hospital data window, reporting an AUROC of 0.811, compared with an AUROC of 0.777 (95% CI 0.764‐0.790) in this study. Differences in performance should be interpreted in the context of substantial methodological variation, including the prediction time horizon, outcome definition, and predictor set. A complementary frailty prediction model developed by Blanes Selva et al [19] achieved an AUROC of 0.89, although that study focused on frailty and mortality prediction to support palliative care assessment and is therefore not directly comparable to acute inpatient frailty screening. Similarly, Zhang et al [20] reported AUROC values ranging from 0.76 to 0.77 using community survey data rather than routinely collected hospital EMR data. Collectively, these findings suggest that the performance of the current model is within the range reported in the literature despite the challenges of early inpatient prediction using data available within 72 hours of admission.

### Interpretation of Key Predictors

The variable importance results confirm known frailty predictors from the literature. Polypharmacy was a key predictor, and the presence of specific medications linked to frailty in the literature was also a key predictor [15]. Laboratory test results for creatinine, albumin, sodium, and the number of blood tests ordered were strong predictors. These specific laboratory tests were derived from the Frailty Index Laboratory Data frailty index, reinforcing the importance of biochemical markers in predicting frailty [8]. AH consult orders emerged as important and previously untested predictors.

The prominence of predictors such as specific medications and abnormal laboratory test results is likely attributable to their direct association with the specific diagnoses underpinning the HFRS score, which was used as the outcome variable. This creates the potential for criterion contamination, whereby the model may partially learn patterns associated with future diagnostic coding and disease burden rather than the broader multidimensional construct of frailty itself. While this does not negate the model’s ability to identify patients likely to receive a frailty classification based on the HFRS, it does mean that model performance should be interpreted as prediction of an HFRS-defined frailty outcome, rather than direct measurement of biological or functional frailty.

Previous research has demonstrated that frailty and multimorbidity are closely related but distinct constructs, with substantial overlap among hospitalized older adults [21]. Consequently, models trained to predict HFRS may identify broader patterns of clinical vulnerability, disease burden, and health care complexity in addition to frailty itself. This may help explain the importance of predictors reflecting multidisciplinary care involvement, polypharmacy, laboratory abnormalities, and health care use within the present model. These findings should therefore be interpreted as predictors of an HFRS-defined frailty phenotype rather than as direct measures of physical or biological frailty alone.

### Value of Multidisciplinary EMR Data for Frailty Prediction

The number of AH consult orders and the presence of specific consult orders, such as speech pathology, occupational therapy, and physiotherapy, were among the strongest predictors of frailty, features not previously identified in frailty prediction models, to our knowledge. This finding suggests that requiring early AH referrals during hospital stay indicates an increased risk of frailty. To our knowledge, our study is the first to demonstrate an association between early AH involvement and frailty risk. Nursing assessment–related variables, including those related to ADL assessments, Braden pressure injury scores, and delirium and cognitive impairment 4-item assessment test scores, were also important predictors. The strong contribution of both nursing-related and AH-related predictors highlights the potential untapped value of multidisciplinary data for frailty and broader health care–related predictive models.

### Timing of Prediction During a Hospital Admission

At 72 hours post admission, the model identified a high proportion of patients with frailty, with high recall (0.873, 95% CI 0.857‐0.887), an AUROC of 0.777 (95% CI 0.764‐0.790), and a *F*_2_-score of 0.755 (95% CI 0.741‐0.768) on the test dataset. The model had good calibration, with an E/O ratio close to 1 (0.996, 95% CI 0.963‐1.028), indicating that the predicted risk probabilities aligned well with observed frailty rates. The model’s performance demonstrates that frailty risk can be reliably estimated early in the hospital stay, using routinely available EMR data, enabling hospital-wide identification of patients at risk of frailty.

Model performance varied across time points according to the amount of clinical data available. Using only data available at 24 hours after admission, the model’s performance declined (*F*_2_-score 0.742, 95% CI 0.725‐0.756; recall 0.827, 95% CI 0.809‐0.844), consistent with limited data completeness early in the admission. By discharge, when clinical documentation was more comprehensive, the model’s performance improved (AUROC 0.835, 95% CI 0.824‐0.846; *F*_2_-score 0.796, 95% CI 0.785‐0.806). This increase in model performance over time supports the conclusion that frailty prediction improves as more clinical data become available. The 72-hour time point provided a pragmatic balance between earlier clinical actionability and greater availability of multidisciplinary data.

### Subgroup Performance and Equity Considerations

Subgroup analysis identified differences in observed frailty rates and model performance by Indigenous status. Overall, Indigenous patients had a lower observed frailty rate than non-Indigenous patients in this cohort (n=531, 29% vs n=8700, 36%), which contrasts with published evidence that frailty is more prevalent among First Nations populations [4]. This difference should be interpreted in the context of differing age distributions and smaller denominators for Indigenous patients, particularly in older age groups. Age-stratified analysis showed similar frailty rates in some younger age bands, with higher frailty among Indigenous patients in the 31 to 40 years (95/346, 28% vs 273/1390, 20%), 51 to 60 years (132/410, 32% vs 924/3255, 28%), 61 to 70 years (117/338, 35% vs 1531/4702, 33%), and 81 to 90 years age groups (11/17, 65% vs 2297/4757, 48%). However, frailty rates were lower among Indigenous patients aged 71 to 80 years (35/121, 29% vs 2180/5609, 39%) and 91 years and older (1/3, 33% vs 774/1431, 54%). These findings indicate that the lower overall frailty rate among Indigenous patients may reflect age structure and limited data, rather than a lower underlying burden of frailty.

Model performance was lower for Indigenous patients than non-Indigenous patients on the *F*_2_-score (0.723, 95% CI 0.664‐0.776 vs 0.757, 95% CI 0.743‐0.770) and recall (0.848, 95% CI 0.784‐0.911 vs 0.874, 95% CI 0.860‐0.890), indicating more missed frailty cases in this subgroup. The AUROC was higher for Indigenous patients (0.798, 95% CI 0.749‐0.846 vs 0.774, 95% CI 0.761‐0.787), while the Brier score was lower (0.152, 95% CI 0.132‐0.172 vs 0.183, 95% CI 0.177‐0.189), suggesting that poorer threshold-dependent classification performance occurred despite comparable or better discrimination and overall probability accuracy. The observation that Indigenous patients had a lower Brier score despite lower recall and *F*_2_-score illustrates the importance of considering multiple model performance measures when evaluating subgroup performance. Unlike recall and *F*_2_-score, which depend on a selected classification threshold, the Brier score evaluates the accuracy of predicted probabilities across all patients. The lower observed frailty prevalence among Indigenous patients in this cohort (n=531, 29% vs n=8700, 36%) may have contributed to lower average predicted probabilities and consequently lower Brier scores, despite poorer threshold-dependent performance. Similar patterns can occur when outcome prevalence differs between groups, highlighting that probability-based measures, discrimination measures, and threshold-dependent classification metrics capture different aspects of predictive performance and should be interpreted together rather than in isolation.

This pattern may reflect differences in frailty prevalence, calibration, threshold suitability, or limitations of the HFRS outcome definition in this population. The HFRS was developed using data from UK hospitals and has not been validated for Indigenous Australians [7]. In addition, *ICD-10*-AM diagnostic patterns used to derive the HFRS may not fully capture frailty-related health in Indigenous patients. These findings support the need for culturally validated frailty measures, subgroup calibration, and external validation in larger Indigenous cohorts.

Model performance was broadly similar between sexes, with only small differences observed across discrimination, classification, and calibration metrics.

### Clinical and Operational Implications

At the selected operating threshold of 0.20, 3360 (62%) admissions were classified as positive for frailty risk. The threshold was intentionally selected to prioritize sensitivity, identifying 87% (1647/1886) of patients with frailty while maintaining a PPV of 49% (1647/3361). Consequently, approximately half of flagged patients would ultimately be classified as frail according to HFRS, while 62% (3360) of admissions would be flagged for review by the model. These trade-offs may be acceptable where the objective is early identification of patients for multidisciplinary frailty assessment rather than definitive diagnosis, although the optimal threshold is likely to depend on local clinical resources and service capacity.

Decision curve analysis demonstrated positive net benefit across all evaluated thresholds, suggesting that use of the model may provide greater clinical utility than reviewing all patients. At the selected threshold of 0.20, 62% (n=3360) of admissions would be identified for potential review. Decision curve analysis estimated a net reduction of 37.5 reviews avoided per 100 admissions compared with a treat-all strategy, after accounting for the trade-off between true-positive and false-positive classifications. These findings suggest that the model may improve targeting of frailty-related review compared with reviewing all patients, particularly where the acceptable intervention threshold lies between 0.10 and 0.30.

Implementation of the model would require consideration of incomplete or unavailable predictor data. During model development, missing categorical values were assigned to an “unknown” category, and missing numeric values were imputed using the training-set mean. In practice, delayed laboratory results, incomplete nursing assessments, or unavailable consult order data could affect prediction accuracy. Operational deployment would, therefore, require ongoing monitoring of data completeness and periodic evaluation of model performance when key predictor variables are missing or recorded outside expected time frames.

The intended use of the model would be as a decision support tool integrated within or alongside the EMR. Predictions would be generated automatically from routinely collected data, without requiring manual data entry by end users. Clinical interpretation and any subsequent intervention would remain the responsibility of treating clinicians and multidisciplinary teams. No specialist expertise in ML would be required of end users; however, appropriate training would be needed to support interpretation of model outputs and integration into existing clinical workflows.

### Limitations of the Study

Key limitations include generalizability and potential subgroup biases. The model was trained only at a single tertiary hospital (RAH), which may limit its generalizability and increase the risk of overfitting, as health care use patterns vary by site.

An increased sample size of Indigenous patients and additional training variables could allow for the development of a model that has better performance for Indigenous patients; however, the lack of a validated frailty index for Indigenous patients makes identifying frailty in Indigenous patients challenging. The incorporation of bias mitigation strategies, such as subgroup calibration, could also potentially improve model performance for Indigenous patients.

A further limitation relates to the use of HFRS as the reference standard. The HFRS was derived from postdischarge ICD-10-AM codes and therefore reflects documented diagnostic burden rather than direct clinical assessment of frailty. Because some predictor variables, particularly laboratory test results and medication use patterns, are associated with diagnoses that contribute to the HFRS calculation, a degree of criterion contamination may exist. Consequently, the model may partially predict future diagnostic coding patterns rather than frailty as a distinct clinical syndrome. Although HFRS is a validated and widely used frailty measure, it may not fully capture functional, cognitive, and social dimensions of frailty that are typically assessed through clinician-administered tools such as the CFS. Future studies should evaluate model performance against alternative frailty measures and prospective clinical assessments.

As HFRS is derived from diagnostic coding data, the outcome may capture broader constructs, including multimorbidity and clinical complexity, in addition to frailty. These constructs frequently overlap in hospitalized patients and may contribute to model performance. Future studies should evaluate model performance against prospective clinical frailty assessments to better distinguish frailty from related dimensions of patient vulnerability and health care complexity.

Data quality is often a challenge when working with EMR data, as it is part of an operational system designed to deliver patient care, not for data analysis. Incomplete and variable-quality data are limitations of this study. Integration of the model into a clinical workflow is a potential future direction, which would require consultation with clinicians. Validation against prospectively collected clinical frailty assessments would also help determine whether the model is identifying frailty itself or predicting the diagnostic burden captured by HFRS.

This study was limited to model development and internal validation. Evaluation of real-world implementation, including alert presentation, workflow integration, clinician acceptance, alert burden, intervention pathways, and patient outcomes, was outside the scope of the current study and should be assessed in prospective implementation studies. Future work should evaluate how model outputs can be incorporated into multidisciplinary frailty pathways and determine the optimal balance between sensitivity, specificity, and service capacity.

### Conclusions

This study developed and validated a frailty prediction model for hospital inpatients using routinely collected EMR data available within 72 hours of admission. The model demonstrated good discrimination (*F*_2_-score of 0.755, 95% CI 0.741‐0.768), high recall (0.873, 95% CI 0.857‐0.887), and reasonable calibration on the test dataset, with E/O ratios close to 1 (0.996, 95% CI 0.963‐1.028), supporting its utility for early frailty identification. Key predictors included polypharmacy, laboratory results (including creatinine, albumin, and sodium), AH consult orders, and nursing assessment data, highlighting the value of multidisciplinary EMR data for frailty risk stratification. Model performance was robust at 72 hours, lower at 24 hours postadmission, and improved further by discharge, indicating that predictive accuracy increases as more data becomes available during a patient’s admission.

Subgroup analysis revealed lower model performance for Indigenous patients, likely reflecting limitations of the HFRS as a frailty score in this population and highlighting the need for culturally validated frailty indices. No substantial differences in model performance were observed between sexes. These findings reinforce the importance of ongoing evaluation of model fairness and subgroup validity.

Future research should prioritize external validation across diverse hospital settings and patient populations to assess generalizability and mitigate overfitting. The development of frailty indices validated for Indigenous Australians and other underrepresented groups is needed to address equity gaps. Integration of the model into clinical workflows, with real-time EMR implementation and clinician engagement, will be essential for translation into practice. Ongoing monitoring for bias and periodic recalibration are recommended to ensure sustained model performance and fairness.

## Supplementary material

10.2196/95478 Multimedia Appendix 1 Final model inputs for XGBoost frailty prediction model.

## Abbreviations

ADLs: activities of daily living
AH: allied health
AUROC: area under the receiver operating characteristic curve
CALHN: Central Adelaide Local Health Network
CFS: Clinical Frailty Scale
E/O ratio: expected-to-observed ratio
EMR: electronic medical record
HFRS: Hospital Frailty Risk Score
ICD-10-AM: International Statistical Classification of Diseases and Related Health Problems, Tenth Revision, Australian Modification
ML: machine learning
NPV: negative predictive value
PPV: positive predictive value
RAH: Royal Adelaide Hospital
ROC: receiver operator curve
SHAP: Shapley additive explanation
SVM: support vector machine
TRIPOD-AI: Transparent Reporting of a multivariable prediction model for Individual Prognosis Or Diagnosis-AI
XGB: XGBoost
