# Supplementary material for: Development and Internal Validation of an Electronic Medical Record–Based Machine Learning Model for Frailty Prediction in Hospitalized Patients: Retrospective Cohort Study
Source: JMIR Med Inform. 2026 Oct 28;14:e95478. doi: 10.2196/95478 (PMC13716358; doi:10.2196/95478)
Supplement: Supplementary file 1 [file medinform-v14-e95478-s001.docx]

## Appendix 1

Final model inputs

| Variable | Description |
| --- | --- |
| AGE | Age on admission |
| BRADEN_MAX | Maximum Braden score at 72 hours |
| CONSULTCOUNT_MED | Number of medical consult orders placed at 72 hours |
| CONSULTCOUNT_AH | Number of allied health consult orders placed at 72 hors |
| DELIRIUM_MAX | Maximum 4AT delirium score at 72 hours |
| MED_COUNT | Number of medications active on medication chart at 72 hours |
| MOVECOUNT | Number of times a patient has moved beds in 72 hours |
| MUST_MAX | Maximum MUST malnutrition score at 72 hours |
| NUMBEROFBLOODS | Number of blood tests ordered at 72 hours |
| ALBUMIN_MIN | Minimum albumin blood test value at 72 hours |
| CREATININE_SERUM_MAX | Maximum creatinine blood test value at 72 hours |
| HAEMOGLOBIN_MIN | Minimum hemoglobin blood test value at 72 hours |
| MCV_MIN | Minimum mean cell volume blood test value at 72 hours |
| SODIUM_LEVEL_MAX | Maximum sodium blood test value at 72 hours |
| SODIUM_LEVEL_MIN | Minimum sodium blood test value at 72 hours |
| OT_CONSULT | Flag for if an occupational therapy consult order has been placed at 72 hours |
| D_CONSULT | Flag for if a dietitian consult order has been placed at 72 hours |
| P_CONSULT | Flag for if a physiotherapy consult order has been placed at 72 hours |
| SP_CONSULT | Flag for if a speech pathology consult order has been placed at 72 hours |
| GENDER | Gender |
| ANXIOLYTIC_SEDATIVE_HYPNOTIC_FLAG | Flag for if an anxiolytic, sedative or hypnotic medication was ordered in the first 72 hours |
| CNS_AGENT_FLAG | Flag for if a CNS agent medication was ordered in the first 72 hours |
| ANTIDEPRESSANT_FLAG | Flag for if an antidepressant medication was ordered in the first 72 hours |
| ASPIRIN_FLAG | Flag for if aspirin was ordered in the first 72 hours |
| ANTIPSYCHOTIC_FLAG | Flag for if an antipsychotic medication was ordered in the first 72 hours |
| VIT_D_FLAG | Flag for if Vitamin D was ordered in the first 72 hours |
| ANTIHYPERLIPIDEMIC_FLAG | Flag for if an antihyperlipidemic medication was ordered in the first 72 hours |
| GERI_CONSULT | Flag for if a geriatrics consult order was placed in the first 72 hours |
| PALLCARE_CONSULT | Flag for if a palliative care consult order was placed in the first 72 hours |
| STROKE_CONSULT | Flag for if a stroke consult order was placed in the first 72 hours |
| CARDIAC_CONSULT | Flag for if a cardiac consult order was placed in the first 72 hours |
| ICU_CONSULT | Flag for if an ICU consult order was placed in the first 72 hours |
| FUNCTIONALLEVEL_TRANSFERS | Assessment of a patient’s functional level for transfers (last recorded value) |
| FUNCTIONALLEVEL_DRESSING | Assessment of a patient’s functional level for dressing (last recorded value) |
| FUNCTIONALLEVEL_COMMUNICATION | Assessment of a patient’s functional level for communication (last recorded value) |
| FUNCTIONALLEVEL_VISION | Assessment of a patient’s functional level for vision (last recorded value) |
| FUNCTIONALLEVEL_AMBULATION | Assessment of a patient’s functional level for ambulation (last recorded value) |
| FUNCTIONALLEVEL_BATHING | Assessment of a patient’s functional level for bathing (last recorded value) |
| FUNCTIONALLEVEL_TOILETING | Assessment of a patient’s functional level for toileting (last recorded value) |
| FUNCTIONALLEVEL_NUTRITIONALINTAKE | Assessment of a patient’s functional level for nutritional intake (last recorded value) |
